# Effect of Itraconazole on the Pharmacokinetics of Diclofenac in Beagle Dogs

**DOI:** 10.3797/scipharm.1003-10

**Published:** 2010-05-19

**Authors:** Fahad I. Al-Jenoobi

**Affiliations:** Department of Pharmaceutics, College of Pharmacy, King Saud University, Riyadh 11451, Saudi Arabia

**Keywords:** Interaction, Pharmacokinetic parameters, Plasma concentration, Absorption, HPLC

## Abstract

The objective of this study was to investigate the potential effect of itraconazole on the pharmacokinetics of diclofenac potassium in beagle dogs after oral coadministration. Five male beagle dogs received a single oral 50 mg dose of diclofenac potassium alone in phase I, and along with a single oral 100 mg dose of itraconazole in phase II. Blood samples obtained for 8.0 hours post dose were analysed for diclofenac concentration using a validated high performance liquid chromatography (HPLC) assay method. The area under plasma concentration-time curve (AUC_0–∞_), maximum plasma concentration (C_max_), time to reach C_max_ (T_max_) and elimination half-life (t_1/2_), were calculated for diclofenac before and after itraconazole administration. The coadministration of itraconazole with diclofenac potassium has resulted in a significant reduction in AUC_0–∞_ and C_max_ of diclofenac, which was about 31 and 42%; respectively. No statistically significant differences were observed for T_max_ and t_1/2_ of diclofenac between the two phases. Therefore, it could be concluded that oral coadministration of itraconazole may have the potential to affect the absorption of diclofenac as indicated by the significant reduction in its AUC and C_max_ in beagle dogs.

## Introduction

Diclofenac is a nonsteroidal anti-inflammatory drug (NSAID) with analgesic and antipyretic properties. It is widely used in management of mild to moderate pain particularly when inflammation is also present as in cases of rheumatoid arthritis, osteoarthritis, musculoskeletal injuries and some postoperative conditions [1–3]. Its pharmacological effects are believed to be due to blocking the conversion of arachidonic acid to prostaglandins by inhibiting cyclo-oxygenase enzymes [4].

Diclofenac is almost completely absorbed after oral administration. However, due to its first-pass hepatic metabolism, only about 50% of the absorbed dose is systematically available [5–8]. The major metabolite of diclofenac in human is 4′-hydroxydiclofenac, which is mainly formed by cytochrome P4502C9 (CYP2C9) enzyme [9–10]. The minor diclofenac metabolites are formed by several enzymes including CYP3A4 [11]. About 99% of the drug is bound to human plasma proteins, mainly albumin [12, 13]. The potassium salt of diclofenac was found to be particularly useful for quick pain relief compared to the sodium salt because of its higher solubility in the stomach acidic medium [14].

Itraconazole is a triazole antifungal agent that is used for a number of indications including systemic and superficial mycosis [15]. It has been shown to interact with many drugs mainly by inhibiting their CYP3A4-mediated metabolism and/or multidrug resistance protein 1 (MRP1)-mediated transport [16–19]. In addition, itraconazole is highly bound to plasma proteins, primarily albumin and therefore, may interact with other albumin highly bound drugs [15, 20].

A literature search revealed that pharmacokinetic interactions between itraconazole and diclofenac have never been investigated. In fact, there is a very limited published information about the pharmacokinetic interactions between triazole antifungals and NSAIDs in general [21]. Coadministration of itraconazole with diclofenac may be considered for the treatment of certain fungal infections in cases of rheumatoid arthritis, musculoskeletal injuries and some postoperative conditions. Therefore, it is important to investigate the potential interaction between the two drugs. The aim of this study was to investigate the effect of itraconazole on the pharmacokinetics of diclofenac in vivo using beagle dogs as an animal model.

## Experimental

### Materials

Diclofenac potassium 50 mg tablets (Cataflam^®^_,_ Novartis Pharma, Egypt) were purchased from the market while itraconazole 100 mg capsules (Sporanox^®^_,_ Janssen-Cilag, Beerse, Belgium) were obtained from the pharmacy of King Khalid University Hospital. Diclofenac and flufenamic acid (internal standard) analytical powders were purchased from Sigma (St. Louis, MO, USA). Acetonitrile was obtained from BDH, England, UK and glacial acetic acid from Polysciences Inc. Warrington, PA, USA. All other reagents were of analytical grade.

### Animal study

The protocol of the in vivo study in beagle dogs was approved by the Experimental Animals Care Centre of College of Pharmacy, King Saud University, Riyadh, Saudi Arabia.

Five healthy male beagle dogs weighing 10.2–13.6 kg were used. The study was conducted in two phases with one week wash-out period. The dogs were fasted for 12 hrs before drug administration and continued fasting for 2 hrs post dose but allowed free access to water. Each dog was administered one tablet of 50 mg diclofenac potassium alone (phase I) or with a 100 mg itraconazole capsule (phase II). No other medications were taken during the study period. Venous blood samples (3.0 ml) were taken from the femoral vein into heparinized tubes before drug administration (to serve as a blank) and at 0.25, 0.50, 0.75, 1.00, 1.50, 2.00, 3.00, 4.00, 6.00 and 8.00 hr after drug administration. Samples were centrifuged immediately at 5000 rpm for 10 min. and the separated plasma samples were kept at −20 °C for analysis.

### Assay of diclofenac in plasma

The plasma concentration of diclofenac was determined by a modified high-performance liquid chromatography (HPLC) assay method [22] using Shimadzu HPLC system (Kyoto, Japan) that is composed of a liquid chromatograph pump (Model LC-20A), a UV detector (Model SPD-20A), a degasser (Model DGU-20A), a communication bus module (Model CBM-20A) and an autosampler (Model SIL-20A). The drug and internal standard were eluted from Nucleosil 5 μm C-18 column (150 mm x 4.6 mm, MACHEREY-NAGEL GmbH & Co. KG, Germany) at an ambient temperature using a mobile phase of acetonitrile and water (50:50 % v/v, adjusted to pH 3.3 with glacial acetic acid) at a flow rate of 1.5 ml/min with a UV detection at 280 nm. To 0.25 ml dog plasma samples, an aliquot of 20 μl internal standard (0.1 μg/ml flufenamic acid) was added followed by shaking on a vortex mixer for 30 sec. Precipitation of serum proteins was achieved by addition of 500 μl cold acetonitrile. The mixture was shaken again on a vortex mixer for 1 min., and centrifuged for 5 min. at 10000 rpm. The supernatant was transferred to an autosampler vial for injection in HPLC.

### Calculation of pharmacokinetic parameters

Maximum plasma concentration (C_max_) and the time to reach it (t_max_) were obtained directly from plasma data. Elimination half-life (t_1/2_) was calculated as 0.693/K_el_, where K_el_ is the elimination rate constant obtained from the slope of the terminal exponential phase. The total area under plasma concentration time curve (AUC_0–∞_) was calculated as the sum of AUC_0–8hr_ and AUC_8hr–∞_, where AUC_0–8hr_ was determined by the trapezoidal rule method and AUC_8hr–∞_ as the last plasma concentration divided by K_el_.

### Statistical analysis

The significance of the differences between plasma concentrations of diclofenac at each sampling time and the pharmacokinetic parameters of treatment group versus control were evaluated using Student’s paired *t*-test. P value ≤ 0.05 was taken as the criterion for statistically significant difference.

## Results and Discussion

**Fig. 1:**
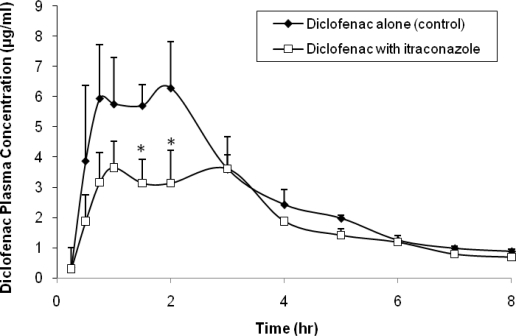
Mean plasma concentration (± SE) of diclofenac in beagle dogs following oral administration of 50 mg diclofenac potassium tablet alone (control) or with 100 mg itraconazole capsule (n=5). *P < 0.05.

Figure 1 shows the plasma concentration of diclofenac in beagle dogs following oral administration of 50 mg diclofenac potassium tablet alone (control) or with 100 mg itraconazole capsule. It was noticed that the plasma concentration of diclofenac was significantly affected by the presence of itraconazole upon oral coadministration to beagle dogs. This finding was supported by the significant reduction in the C_max_ and AUC_0–∞_ after itraconazole administration (about 42% and 31%; respectively, Table 1). No statistically significant differences (P > 0.05) were observed for the values of t_max_ and t_1/2_ after itraconazole treatment compared to control, which indicated similar times to reach maximum concentration and similar elimination rate constants.

Itraconazole is a known inhibitor of CYP3A subfamily of enzymes and most of the reported pharmacokinetic interactions that are caused by this drug are believed to be mainly due to inhibition of CYP3A. However, the observed interaction in this study can not be explained by inhibition of drug metabolism simply because such inhibition would result in an increase in the AUC and plasma concentration of the affected drug rather than a decrease as observed with diclofenac in this study. In fact, CYP3A subfamily is not a major contributor to diclofenac elimination both in human and in beagle dog [5, 6, 9, 10]. In addition, alteration of diclofenac elimination, in general, does not seem to play a major role in the observed interaction as strongly indicated by the lack of significant itraconazole effect on the t_1/2_ of diclofenac [Table 1].

Displacement of diclofenac from its plasma binding sites by itraconazole is unlikely to be a major cause for this observed interaction. This is supported by the finding of Hynninen and colleagues who have shown that itraconazole could significantly reduce the C_max_ and AUC_0–∞_ of meloxicam in human subjects without affecting the unbound plasma meloxicam concentration [21].

The results obtained from this study suggest that itraconazole reduces the plasma concentration of diclofenac by interfering with its gastrointestinal absorption. Impairment of absorption by itraconazole has been suggested for the first time by Hynninen and coauthors to explain itraconazole effect on the plasma concentration of another NSAID; meloxicam [21]. However, the exact mechanism of such interaction is not clear. Appropriate mechanistic studies that involve suitable intestinal absorption models may help to clarify the mechanism(s) behind this interaction.

In conclusion, itraconazole was shown in this study to significantly reduce the C_max_ and AUC_0–∞_ of diclofenac in beagle dogs suggesting that it has the potential to decrease the intensity of diclofenac pharmacological effect. The exact mechanism of this interaction is not clear but the results suggest an alteration in diclofenac absorption by itraconazole. In addition, results obtained from this study warrant further investigation in human subjects to evaluate the clinical relevance of this interaction.

## Figures and Tables

**Table 1:** Pharmacokinetic parameters of diclofenac in beagle dogs (mean ± SD) following oral administration of 50 mg diclofenac potassium tablet alone (control) or with 100 mg of itraconazole capsule (n=5). *P < 0.05.

**Pharmacokinetic Parameter**	**Diclofenac Alone (control)**	**Diclofenac with Itraconazole**
C_max_ (μg/ml)	6.28 ± 3.46	3.65 ± 2.22*
t_max_ (h)	1.15 ± 0.60	1.94 ± 1.23
AUC_0–∞_ (μg.h/ml)	25.43 ± 6.18	17.65 ± 5.09*
t_1/2_ (h)	2.10 ± 0.37	1.98 ± 0.07
